# International medical graduates pursuing pediatric neurosurgery fellowship training in North America: a 30-year analysis

**DOI:** 10.1007/s00381-026-07169-0

**Published:** 2026-02-13

**Authors:** Alexandria Sorensen, Logan Muzyka, Bianca Luna-Lupercio, Susan Durham, Natalie R. Limoges

**Affiliations:** 1 University of California San Francisco School of Medicine, San Francisco, CA USA; 2 The University of Washington, Seattle, WA USA; 3 USC Medical School, Children’s Hospital of Los Angeles, Los Angeles, CA USA; 4 Rainbow Babies Hospital, Case Western Reserve University, Cleveland, OH USA

**Keywords:** IMG, Pediatric Neurosurgery, Workforce

## Abstract

**Purpose:**

Internati onal medical graduates (IMGs) comprise 25% of practicing physicians in the USA, but their representation in the pediatric neurosurgical workforce has yet to be explored. This study evaluates the impact of IMG status on fellowship match rates, board certification rates, and practice locations among individuals completing pediatric neurosurgical fellowships in the USA from 1983 to 2023.

**Methods:**

Graduates of accredited fellowships were identified using a database maintained by the Accreditation Council for Pediatric Neurosurgery Fellowships (ACPNF). Demographics, medical school, residency/fellowship programs, current practice location, and ABNS and APBNS certification status were determined through internet searches. Graduates were categorized by IMG status, defined as having graduated from a medical school outside of the USA and Canada.

**Results:**

Of the 488 graduates of accredited fellowships from 1992 to 2023, 17% (82) were IMGs. IMGs have lower rates of ABNS and ABPNS certification compared to USMGs (ABNS IMG 24.7%, USMG 74.7%, *p* < 0.001; ABPNS IMG 14.3%, USMG 62.9%, *p* < 0.001). IMGs are more likely to practice outside of the USA compared to USMGs (IMGs 55.8%, USMG 9.0%, *p* < 0.001). IMGs have significantly lower fellowship match rates compared to USMGs (69.5% IMG, 98.5% USMG,* p* < 0.001).

**Conclusion:**

Over the last decade, the percentage of IMGs completing pediatric neurosurgical fellowship has declined significantly. IMG fellows have lower rates of ABNS and APBNS certification and were less likely to practice in the USA compared to USMGs. These findings highlight the diminishing role of IMGs in the US pediatric neurosurgical workforce, raising concerns about the capacity to address global pediatric neurosurgical shortages.

## Introduction

In 2012, the American Association of Neurological Surgeons issued a statement about a growing concern regarding a shortage of pediatric neurosurgeons in the twenty-first century [1]. While the number of Accreditation Council for Pediatric Neurosurgical Fellowships (ACPNF)–accredited pediatric neurosurgical fellowship training positions has steadily increased in the past two decades, many regions, especially those designated as Health Professional Shortage Areas (HPSA) by the US government, continue to face limited access to specialty care [2]. Globally, nearly two-thirds of the population lacks access to adequate surgical care, underscoring the need for innovative solutions to workforce shortages [3]. International medical graduates (IMGs) represent a subset of the pediatric neurosurgical workforce capable of addressing these shortages, both domestically and internationally.

IMGs, defined as physicians who graduated from a medical school outside of the USA and Canada, constitute 25% of all practicing physicians in the country. However, IMGs face unique challenges, including the need for Educational Commission for Foreign Medical Graduates (ECFMG) certification and visa requirements to be eligible for fellowship training programs within the USA [4]. Prior to 2021, the most common method for IMGs to begin training in the USA is through a J-1 visa, which permits graduate medical training throughout residency or fellowship [4]. However, the J-1 visa program requires recipients to leave the USA for 2 years after completing residency training before they can apply for re-entry into the country for fellowship training or practice [4]. This exit requirement may be waived if IMGs agree to practice in an HPSA-designated region [4]. As such, IMGs trained under this policy may play an important role in addressing pediatric neurosurgical care shortages, particularly in underserved areas, while helping to combat the previously anticipated shortage of pediatric neurosurgeons. Since 2021, the ECFMG stopped issuing J-1 visas for non-standard training programs not directly accredited by the ACGME, including pediatric neurosurgery fellowship [5]. Under this new policy, pediatric neurosurgery fellowship programs must be located at institutions willing to sponsor H1b and O-visas for trainees directly [5].

Despite their critical role, little is known about the career trajectories of IMGs who complete pediatric neurosurgical fellowships, including their board certification rates and practice locations. This study aims to evaluate the impact of IMG status on pediatric neurosurgery fellowship match rates, American Board of Neurological Surgery (ABNS) and American Board of Pediatric Neurological Surgery (ABPNS) certification rates, and practice locations compared to USMGs. This information will provide essential insights into the future composition of the global pediatric neurosurgical workforce and highlight opportunities to strengthen support for IMGs in addressing global surgical care disparities.

## Methods

### Identification of Pediatric Neurosurgical Fellowship graduates

From a database maintained by the ACPNF, names of graduates of accredited fellowships and the year of graduation were identified from 1983 to 2023. This database, along with supplemental internet searches, was used to determine gender, medical school, residency training program, fellowship program, residency and fellowship graduation year, and current practice location of each individual. Board certifications for each graduate were determined by conducting a name search in the internet databases maintained by ABNS and APBNS on their respective websites.

### Classification and statistical comparison of IMGs and USMGs

Graduates were categorized as IMGs or USMGs (IMGs having graduated from a medical school outside of the USA) and their medical school country noted. Individuals were further categorized based on whether they completed residency outside the USA. For the purpose of this analysis, medical school graduates from Canada were grouped with the USMGs due to board eligibility and congruence between training programs in Canada and the USA. Graduates were defined as “underrepresented in medicine (URM)” per AAMC’s definition, which excludes white and Asian-identifying graduates. Current practice locations were identified and those within the USA located by zip code. Current practices were further categorized based on setting with the distinctions: Freestanding Children’s Hospital, Academic; Freestanding Children’s Hospital, private or community; Academic Hospital within a Hospital; Private or Community with Pediatric Services; Other; Retired/Deceased/Unable to Locate. Graduates were grouped by decade: 1992–2000, 2001–2010, and 2011–2020. For this portion of the analysis, graduates prior to 1992 were excluded, as ACPNF officially began accepting fellows that year. Current trainees were identified and excluded from relevant analyses. Differences between groups were assessed using chi-square analyses for categorical variables and independent samples *t*-tests for continuous variables. For comparison of more than two groups, additional post-hoc analysis via standard adjusted residual testing were performed, with values >|1.96| denoting significance. Significance was set at *p* < 0.05. Statistical testing was performed using IBM SPSS Statistics for Macintosh (version 28.0.1.1) (Armonk, NY).

## Results

Five hundred and twenty-five applicants to ACPNF fellowships were identified, 488 (91.9%) of whom graduated from fellowship. For the bulk of the analysis, only those who graduated from fellowship were included. Based on the previously described criteria, 406 (83.2%) were classified as USMGs and 82 (16.8%) were classified as IMGs.

### Demographics and training of IMGs versus USMGs

Among IMGs, there were significantly fewer female graduates (*p* < 0.001); however, more IMGs identified with underrepresented races/ethnicities in medicine (*p* = 0.014), per AAMC criterion (Table 1).

**Table 1:** Comparison by medical graduation status, fellowship graduates only

Variable	IMG	USMG	*P* value
Number of responses	82	406	
Demographics			
Female sex	9 (11.0)	119 (29.3)	< 0.001
Race and ethnicity			< 0.001
Asian	26 (31.7)^§^	70 (17.3)^§^	
Black/African American	3 (3.7)	17 (4.2)	
Hispanic/Latinx	10 (12.2)^§^	12 (3.0)^§^	
White	43 (52.4)^§^	307 (75.6)^§^	
Underrepresented in medicine	13 (15.9)	30 (7.4)	0.014
US or CAN residency	34 (41.5)	406 (100.0)	
Other residency	48 (58.5)	0 (0.0)	
ABNS certified*	19 (24.7)	272 (74.7)	< 0.001
ABPNS certified*	11 (14.3)	229 (62.9)	< 0.001
Fellowship			
Top 10 fellowship program	8 (9.8)	150 (36.9)	< 0.001
Practice type			
Practice within the USA**	34 (44.2)	345 (91.0)	< 0.001
Academic practice**	60 (77.9)	260 (68.6)	0.103

Chi-square analyses were performed with significance set at 95% confidence interval

*Excluding candidates who are not yet eligible at the time of data collection. IMG (*n* = 5), USMG (*n* = 42)

**Excluding current trainees at the time of data collection. IMG (*n* = 5), USMG (*n* = 27)

^§^Additional standard adjusted residual testing >|1.96|, indicating significance

IMGs had a significantly longer interval between graduating from medical school and applying into fellowship training, averaging at 12.31 ± 5.59 years for IMGs and 8.08 ± 1.54 for USMGs (*p* < 0.001, 95% CI |−4.87–3.59|). Of the 82 IMG fellowship graduates, 29 (35.4%) have completed residency in the USA or Canada and are thus board eligible. IMGs were significantly less likely than USMGs to hold ABNS or ABPNS certification than USMGs (*p* < 0.001) (Table 1).

### Practice characteristics of IMGs versus USMGs

IMGs were significantly less likely to attend a fellowship program at a children’s hospital deemed Top 10 by the US News and World Report in comparison to USMGs (*p* < 0.001) [6]. Following completion of an ACPNF accredited fellowship, IMGs are also less likely than USMGs to practice within the USA (< 0.001) (Table 1).

### Comparison by decade from 1992 to 2020

The number of IMGs pursuing pediatric neurosurgical fellowships increased significantly after 2000, rising from 10.5% in 1992–2000 to 26.5% in 2001–2010, before declining to 12.0% in 2011–2020 (*p* < 0.001) (Table 2).

**Table 2:** Comparison by decade from 1992 to 2020

Variable	1992–2000	2001–2010	2011–2020	*P* value
Number of responses	76	170	242	
Demographics				
Female sex	12 (15.8)^§^	29 (17.1)^§^	87 (35.9)^§^	< 0.001
IMG	8 (10.5)^§^	45 (26.5)^§^	29 (12.0)	< 0.001
Race and ethnicity				0.014
Asian	5 (6.6)^§^	44 (26.0)^§^	47 (19.4)	
Black/African American	1 (1.3)	9 (5.3)	10 (4.1)	
Hispanic/Latinx	4 (5.3)	7 (4.1)	11 (4.5)	
White	66 (86.8)^§^	110 (64.7)^§^	174 (71.9)	
Underrepresented in medicine	5 (6.6)	17 (10.0)	21 (8.6)	0.677
ABNS certified*	59 (77.6)^§^	117 (68.8)	115 (58.7)^§^	0.007
ABPNS certified*	45 (59.2)	86 (50.6)	109 (55.6)	0.403
Fellowship application				
Top 10 fellowship program	22 (27.2)	50 (29.4)	88 (36.4)	0.739
Current practice type				
Practice within the USA**	58 (76.3)	131 (77.1)	190 (90.0)	< 0.001
Academic practice**	53 (69.7)	112 (65.9)	156 (73.9)	0.231

Chi-square analyses were performed with significance set at 95% confidence interval

*Excluding candidates who are not yet eligible at the time of data collection (*n* = 47)

**Excluding current trainees at the time of data collection (*n* = 32)

^§^Additional standard adjusted residual testing >|1.96|, indicating significance

### Comparison of fellowship match rates

Among the 525 applicants in the cohort, unmatched or withdrawn applicants were significantly more likely to be IMGs (*p* < 0.001). The proportion of applicants successfully matching into fellowship declined in 2011–2020 compared to previous decades, with 11.1% going unmatched and 2.1% withdrawing from the process (*p* < 0.001). Notably, all unmatched and withdrawn applicants in this period were from the 2011–2020 cohort (Table 3).

**Table 3:** Comparison by fellowship match status, by *applicant* cohort

Variable	Fellowship match	Unmatched	Withdrawn application	*P* value
	488	31	6	
Comparison by medical graduation status				
Medical graduation status				< 0.001
IMG	82 (16.8)^§^	25 (80.6)^§^	4 (66.7)^§^	
USMG	406 (83.2)^§^	6 (19.4)^§^	2 (33.3)^§^	
Comparison by decade				
Decade				< 0.001
1992–2000	76 (15.5)^§^	0^§^	0	
2001–2010	170 (34.8)^§^	0^§^	0	
2011–2020	243 (49.7)^§^	31 (100.0)^§^	6 (100.0)^§^	

Chi-square analyses were performed with significance set at 95% confidence interval

^§^Additional standard adjusted residual testing >|1.96|, indicating significance

### IMGs current practice

There are 37 of 82 (45.1%) IMGs currently practicing outside of the USA and Canada. Of the 37, 78.4% (*29/37*) practice in the same country where they earned their medical degree, while 21.6% (8/37) practice internationally in a different country than they earned their medical degree.

### IMGs by region

All continents for the exception of Antarctica are represented among the 82 IMG graduates of ACPNF accredited fellowships. Six (7.3%, 6/82) attended medical school in South America, 33 (40.2%, 33/82) attended medical school in Asia, 8 (9.8%, 8/82) attended medical school in Africa, 6 (7.3%, 6/82) attended medical school in North America outside of the US (including Caribbean), 2 attended medical school in Australia (2.4%, 2/82), and 27 (32.93%, 27/82) attended medical school in Europe.

Graduates from medical schools in the Middle East—Bahrain, Israel, Jordan, Lebanon, Pakistan, Saudi Arabia, United Arab Emirates, Egypt, and Iraq—were classified by geographic location into Asia and Africa.

## Discussion

This study demonstrates the disparity between matching to pediatric neurosurgical fellowship as an IMG as opposed to as a USMG. The decline in IMGs entering pediatric neurosurgical fellowships over the last decade may exacerbate global workforce shortages in pediatric neurosurgery. Despite a threefold increase in pediatric neurosurgery fellowship programs over the past 30 years, the rise in USMG applicants has likely contributed to the decline in IMG representation demonstrated by this study. The change in visa types and sponsoring bodies, placing the burden upon the educational institutions directly, also complicates the acceptance and training of IMG applicants. With significant cost and bureaucracy to obtaining licensure and visas, and varied eligibility of licensure across states, this disincentivizes programs from matching an IMG candidate [7]. Since USMG fellows are more likely to remain in the USA after training, the increase in the number of fellowship-trained pediatric neurosurgeons does little to address the international shortage of pediatric neurosurgeons, leaving many patients with treatable neurosurgical conditions without access to care [3]. Given their higher likelihood of practicing outside the USA after fellowship, IMGs play a crucial role in expanding the global scope of pediatric neurosurgery, underscoring the need for strategies that support their participation in training programs.

### Gender in IMGs

The decline in IMGs entering pediatric neurosurgical fellowships not only impacts global workforce shortages but also disproportionately affects women. Women remain significantly underrepresented among IMG fellowship graduates, comprising only 11.0% of the population compared to 29.3% of USMG fellowship graduates (Table 1). This trend mirrors that of the broader neurosurgical workforce, with women comprising only 19% of the field and even as little as 5% in specialties such as spine surgery [8, 9]. These disparities are rooted in systemic challenges, including biases in mentorship, limited leadership opportunities, and societal expectations regarding work-life balance [8, 10, 11]. Additionally, in regions with limited educational access for women, systemic barriers may contribute to the underrepresentation of women IMGs in pediatric neurosurgical fellowships [11, 12]. Addressing these barriers requires proactive initiatives such as international mentorship programs, targeted outreach, and structural changes in training pathways to support and encourage female IMGs in pursuing pediatric neurosurgical fellowships. Expanding these efforts could help diversify the global neurosurgical workforce and improve access to care.

### Board certification, fellowship placement, and academic practice

The disparity in board certification rates between IMGs and USMGs likely reflects structural barriers rather than differences in competency. ABNS eligibility requires an ACGME-accredited residency, inherently disqualifying IMGs completing their residency training abroad [13]. However, among ABNS-eligible IMGs, those that completed medical school outside of the USA but completed an ACGME residency, certification rates match those of USMGs, indicating that residency training location—not IMG status—drives this disparity. In contrast, lower ABPNS certification rates among IMGs likely stem from its limited value internationally and the prevalence of mixed adult-pediatric practices outside the USA [14]. While this manuscript focuses on end points of IMG certification in the USA, it is important to note that IMGs who train in Canada may pursue board certification by the Royal College of Physicians and Surgeons as an equivalent credential. Those with Royal College of Physicians and Surgeons recognition may also be eligible for ABPNS certification through the ABPNS-RCPSC dual sub-specialty credential after completing an ABPNS-accredited fellowship [15]. While there are pathways for IMGs to attain ABPNS certification, depending on their post-residency training, they may be required to complete additional training compared to their USMG colleagues to practice within the USA [15]. These findings underscore how certification requirements influence workforce distribution and highlight how alternative credentialing pathways can support global pediatric neurosurgical care.

Beyond certification, IMGs also face structural and systemic barriers in securing competitive fellowships and academic positions [16]. VISAs, state varied licensure, and work permit complexities create logistical and financial hurdles, while limited administrative support in the international physician onboarding process further restricts opportunities. These administrative burdens may contribute to implicit and explicit biases in selection processes, making it more difficult for IMGs to access top-tier training programs and faculty roles [17, 18]. These challenges underscore the need for policies that streamline visa processes, enhance institutional support, and promote equitable evaluation criteria to ensure that highly qualified IMGs can fully contribute to the academic neurosurgical workforce.

### Practice location

Almost half of all IMGs applying to pediatric neurosurgery fellowships originate from well-resourced regions such as Asia and Europe, while representation from areas with significant neurosurgical care shortages—such as Africa and South America—remains low [3]. Given that over half of IMG graduates return to their home countries after fellowship, expanding IMG training pathways presents a critical opportunity to enhance pediatric neurosurgical capacity in underserved regions.

Despite a threefold increase in fellowship programs, the number of IMG trainees has declined over the past decade, likely due to increasing competition from USMGs and visa complexities. This trend reduces the number of fellowship-trained pediatric neurosurgeons practicing internationally, exacerbating workforce shortages and limiting access to life-saving neurosurgical care [3]. As of 2018, only 330 of the approximately 2200 pediatric neurosurgeons practicing globally were located in low- and lower-middle-income countries [19]. These findings emphasize the need for further training and infrastructure support in these regions. Encouragingly, studies also indicate widespread interest among neurosurgeons in global collaboration, underscoring the potential for international training initiatives to address these gaps [20, 21].

Efforts to strengthen the pediatric neurosurgical workforce should include recruiting both USMG and IMG-trained specialists for global initiatives while also increasing outreach to IMGs from underserved regions. Expanding awareness of US-based fellowship opportunities, streamlining pathways for IMGs to train in high-need areas, and investing in long-term partnerships can help mitigate disparities and improve global pediatric neurosurgical care [3].

### Limitations

While these data allow us to preliminarily characterize the population of pediatric neurosurgical fellows entering the international workforce, there are limitations to the study which stand to be addressed in further research. Some demographic and practice data were obtained from publicly available institutional websites and US News & World Report profiles, limiting accuracy, particularly for physicians practicing outside the USA and Canada. Efforts to allow pediatric neurosurgical fellowship graduates to self-identify race, sex, and practice characteristics to further validate the results described are currently underway. Similarly, gathering survey-based data on the motivations for IMGs to train in the USA, reasons for moving their practices abroad or staying in the USA, and barriers to working or training in the USA could elucidate important areas of inquiry for the neurosurgical community to help bolster inclusivity of IMGs and expand the number of US-trained pediatric neurosurgeons entering the global work force.

## Conclusions

The decline in IMGs entering pediatric neurosurgical fellowships, despite an increase in available fellowship programs, highlights systemic barriers that may exacerbate global workforce shortages. Board certification requirements, visa complexities, and institutional biases likely contribute to disparities in training opportunities and academic advancement for IMGs. Addressing these challenges through policy reforms, mentorship initiatives, and expanded international training pathways is essential to strengthening the global pediatric neurosurgical workforce. Future efforts should focus on improving accessibility for IMGs from underserved regions to enhance neurosurgical care worldwide.

## Data Availability

The data that support the findings of this study were obtained from the Accreditation Council for Pediatric Neurosurgery Fellowships (ACPNF) and are not openly available due to reasons of sensitivity. The database is available from the senior author, Dr. Limoges, upon reasonable request. The database is located in controlled access data storage with the ACPNF.

## References

[1] Statement of the American Association of Neurological Surgeons on the subject of ensuring an adequate neurosurgical workforce for the 21st century. December 19, 2012. https://www.aans.org/-/media/Files/AANS/Advocacy/2012-News/NeurosurgeryIOMGMEPaper121912.ashx. Accessed 18 Oct 2023

[2] Arredondo K, Touchett HN, Khan S, Vincenti M, Watts BV (2023) Current programs and incentives to overcome rural physician shortages in the United States: a narrative review. J Gen Intern Med 38(3):916–922. 10.1007/s11606-023-08122-6 37340266 10.1007/s11606-023-08122-6 PMC10356718

[3] Dewan MC, Rattani A, Fieggen G et al (2018) Global neurosurgery: the current capacity and deficit in the provision of essential neurosurgical care. Executive Summary of the Global Neurosurgery Initiative at the Program in Global Surgery and Social Change. J Neurosurg 130(4):1055–1064. 10.3171/2017.11.JNS171500 10.3171/2017.11.JNS171500 29701548

[4] Hart LG, Skillman SM, Fordyce M, Thompson M, Hagopian A, Konrad TR (2007) International medical graduate physicians in the United States: changes since 1981. Health Aff 26(4):1159–1169. 10.1377/hlthaff.26.4.1159 10.1377/hlthaff.26.4.1159 17630460

[5] Recognition of non-standard training for exchange visitor (J-1) physicians to transition to the ACGME. ACGME. April 19, 2021. https://www.acgme.org/newsroom/2021/4/recognition-of-non-standard-training-for-exchange-visitor-j-1-physicians-to-transition-to-the-acgme/. Accessed 2 Jan 2026

[6] Best Children’s Hospitals 2023–2024 honor roll and overview. US News & World Report. https://health.usnews.com/health-news/best-childrens-hospitals/articles/best-childrens-hospitals-honor-roll-and-overview. Accessed 23 Oct 2023

[7] FSMB | State specific requirements for initial medical licensure. fsmb. https://www.fsmb.org/step-3/state-licensure/. Accessed 2 Jan 2026

[8] Garozzo D, Rispoli R, Graziano F et al (2022) Women in neurosurgery: historical path to self-segregation and proposal for an integrated future. Front Surg 9:908540. 10.3389/fsurg.2022.908540 35836607 10.3389/fsurg.2022.908540 PMC9274114

[9] Muzyka L, Pugazenthi S, Lavadi RS et al (2023) Geographic distribution in training and practice of academic neurological and orthopedic spine surgeons in the United States. World Neurosurg 176:e281–e288. 10.1016/j.wneu.2023.05.050 37209918 10.1016/j.wneu.2023.05.050

[10] Odell T, Toor H, Takayanagi A et al Gender disparity in academic neurosurgery. Cureus 11(5):e4628. 10.7759/cureus.4628 10.7759/cureus.4628 PMC6623992 31312554

[11] Koutsouras GW, Zhang L, Zanon N, Lam S, Boop FA, Tovar-Spinoza Z (2022) Equity in neurosurgery: a worldwide survey of women neurosurgeons. J Neurosurg 138(2). https://thejns.org/view/journals/j-neurosurg/138/2/article-p550.xml. Accessed 25 Dec 2024 10.3171/2022.6.JNS22466 35907187

[12] Shi HH, Westrup AM, O’Neal CM, Hendrix MC, Dunn IF, Gernsback JE (2021) Women in neurosurgery around the world: a systematic review and discussion of barriers, training, professional development, and solutions. World Neurosurg 154:206-213.e18. 10.1016/j.wneu.2021.07.037 34280544 10.1016/j.wneu.2021.07.037

[13] Norcini JJ, Boulet JR, Whelan GP, McKinley DW (2005) Specialty board certification among U.S. citizen and non-U.S. citizen graduates of international medical schools. Acad Med 80(10 Suppl):S42-45. 10.1097/00001888-200510001-00014 16199456 10.1097/00001888-200510001-00014

[14] Nadel JL, Scott RM, Durham SR, Maher CO (2019) Recent trends in North American pediatric neurosurgical fellowship training. J Neurosurg Pediatr 23(4):517–522. 10.3171/2018.10.PEDS18106 30611157 10.3171/2018.10.PEDS18106

[15] ABPNS Certification. Certification. https://abns.org/abpns-certification/. Accessed 2 Jan 2026

[16] Chen PGC, Curry LA, Bernheim SM, Berg D, Gozu A, Nunez-Smith M (2011) Professional challenges of non-U.S.-born international medical graduates and recommendations for support during residency training. Acad Med 86(11):1383–1388. 10.1097/ACM.0b013e31823035e1 21952056 10.1097/ACM.0b013e31823035e1 PMC3257160

[17] Desbiens NA, Vidaillet HJ (2010) Discrimination against international medical graduates in the United States residency program selection process. BMC Med Educ 10:5. 10.1186/1472-6920-10-5 20100347 10.1186/1472-6920-10-5 PMC2822781

[18] Smith SM, Parkash V (2023) Normalized “medical inferiority bias” and cultural racism against international medical graduate physicians in academic medicine. Acad Pathol 10(4):100095. 10.1016/j.acpath.2023.100095 37767366 10.1016/j.acpath.2023.100095 PMC10520300

[19] Dewan MC, Baticulon RE, Rattani A, Jr. JMJ, Warf BC, Harkness W (2018) Pediatric neurosurgical workforce, access to care, equipment and training needs worldwide in: Neurosurgical Focus Volume 45 Issue 4 Journals. https://thejns.org/focus/view/journals/neurosurg-focus/45/4/article-pE13.xml?tab_body=fulltext. Accessed 14 Sept 2024 10.3171/2018.7.FOCUS18272 30269579

[20] Fuller AT, Barkley A, Du R et al (2020) Global neurosurgery: a scoping review detailing the current state of international neurosurgical outreach. 10.3171/2020.2.JNS192517 10.3171/2020.2.JNS192517 32384268

[21] Lepard JR, Akbari SHA, Haji F, Davis MC, Harkness W, Johnston JM (2020) The initial experience of InterSurgeon: an online platform to facilitate global neurosurgical partnerships. Neurosurg Focus. 10.3171/2019.12.FOCUS19859 32114551 10.3171/2019.12.FOCUS19859

